# Supplementary figures and images for: Investigation of the Pathogenic Mechanism of Ciprofloxacin in Aortic Aneurysm and Dissection by an Integrated Proteomics and Network Pharmacology Strategy
Source: J Clin Med. 2023 Feb 6;12(4):1270. doi: 10.3390/jcm12041270 (PMC9967027; doi:10.3390/jcm12041270)

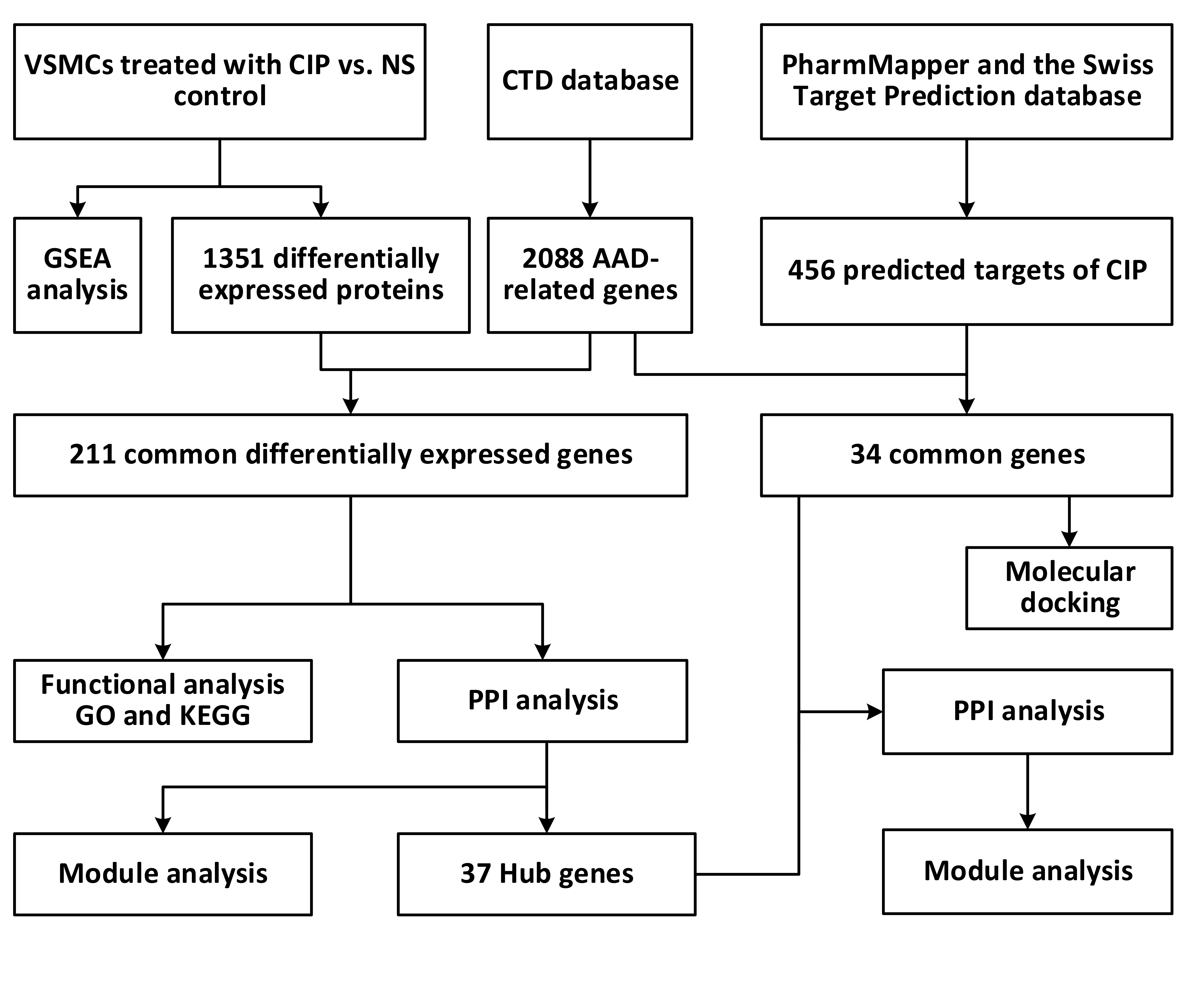

Supplement: Supplementary file 1 [file jcm-12-01270-s001.zip › Figure S1.tif]
